# Using existing cohorts in research on environmental causes of diseases – pros and cons from a European Perspective

**DOI:** 10.1186/1753-6561-7-S5-O9

**Published:** 2013-08-30

**Authors:** N Kuenzli

**Affiliations:** 1 Medical Faculty University Basel, Basel, Switzerland; 2 Department of Epidemiology and Public Health, Swiss Tropical and Public Health Institute (SwissTPH), Switzerland

## 

Cohorts are an excellent method to study the role of environmental factors in causing chronic diseases, but usually takes decades of data collection. Further studying environmental causes of diseases requires assessment of environmental exposures. However many environmental exposures can be assessed independently from study subjects by retrospective characterization using existing monitoring data and modeling. Therefore it is tempting to add retrospective and prospective environmental exposure assessment to existing cohorts for studying the role of such factors in causation of chronic diseases.

An example of such studies is the ESCAPE (European Study of Cohorts for Air Pollution Effects) which is a large inter-disciplinary collaboration of 25 European partners in 17 countries linking 32 existing cohort studies including the SAPALDIA cohort in Switzerland. It aims to characterize long term exposure to traffic related pollution and its association with chronic pathologies with particular reference to pregnancy & birth outcomes, respiratory diseases, cardiovascular diseases and cancer. The work package for respiratory health in adults aims to investigate the effect of ambient air pollution on the level and change of lung function (FVC and FEV1), prevalence and incidence of COPD, prevalence of chronic bronchitis symptoms and incidence of asthma. Such studies using existing cohorts are faster, cheaper and efficient due to availability of long term health information including early life time covariates, socio-economic information, past changes in health and known co-factors. Availability of different phenotypes and biobanks are also advantageous. If environmental monitoring data are available it is easy to link with existing cohort data. However the major disadvantage will be the heterogeneity of study designs with respect to population selection, sampling, age structure etc. Large heterogeneity can occur with the questionnaires used, the health assessment methods, phenotype definitions, biobank markers, and quality of retrospective exposure assessment. Therefore “other-purpose” existing cohorts are complementary but not an alternative to establishing fully standardized cohorts, biobanks and enviro-banks. Large scale new mega cohorts with biobanks and enviro-banks have major scientific advantages that cannot be accomplished with uncoordinated smaller cohorts.

